# DYSTROPHIC CALCIFICATION OF MAXILLARY SINUS IN PEDIATRIC PATIENTS WITH LIVER TRANSPLANTATION AND PIGMENTATION OF DENTAL ORGAN

**DOI:** 10.1590/1984-0462/;2018;36;1;00012

**Published:** 2017-11-17

**Authors:** Adriana Furtado de Macedo, Claudio Costa, Regina Helena Guedes da Motta Mattar, Ramiro Anthero de Azevedo

**Affiliations:** a Escola Paulista de Medicina, Universidade Federal de São Paulo, São Paulo, SP, Brasil.; b Universidade Cruzeiro do Sul, São Paulo, SP, Brazil.; c Universidade de São Paulo, São Paulo, SP, Brasil.

**Keywords:** Adolescent, Liver transplantation, Maxillary sinus, Pigmentation, Dentition, permanent, Tomography, X-ray computed, Adolescente, Transplante de fígado, Seio maxilar, Pigmentação, Dentição permanente, Tomografia computadorizada por raios X

## Abstract

**Objective:**

To report a case of severe dystrophic calcification in maxillary sinus of a child with liver transplantation and dental organs pigmented by hyperbilirubinemia.

**Case description:**

female patient, 12 years old, with liver transplantation performed at the age of 7 due to extrahepatic biliary atresia (EHBA). The patient was receiving the immunosuppressant tacrolimus (2 mg daily). Intraoral clinical exam showed tooth green pigmentation by bilirubin. Cone-beam volumetric computed tomography (CT) was performed to verify radiographic density of pigmented dental elements. Hounsfield scale measurement did not show changes in radiographic density of dental structures. However, CT scan showed intense dystrophic calcification in the maxillary sinus region.

**Comments:**

CT scan indicated relevant radiographic findings, with radiopacity of the maxillary sinus due to fungal or non-fungal sinusitis. This case report highlights the presence of radiographic image associated with acute infectious processes that could compromise the systemic state of immunosuppressed patients.

## RESUMO

**Objetivo:**

Relatar um caso de calcificação distrófica intensa no interior do seio maxilar em uma criança com transplante hepático e órgãos dentais pigmentados por hiperbilirrubinemia.

**Descrição do caso:**

Paciente do sexo feminino, 12 anos de idade, com transplante hepático efetuado aos 7 anos de vida devido à atresia de vias biliares extra-hepática, uso de tacrolimus imunossupressor (2 mg diários). No exame clínico intrabucal, observou-se a presença de pigmentação esverdeada no órgão dental por bilirrubina. Efetuou-se um exame de tomografia computadorizada volumétrica de feixe cônico para análise da densidade radiográfica dos elementos dentais pigmentados. Mediante interpretação da imagem pela escala de Hounsfield, não foi constatada nenhuma alteração na densidade radiográfica das estruturas do órgão dental. No entanto, a tomografia computadorizada evidenciou a presença de calcificação distrófica intensa em região de seio maxilar.

**Comentários:**

A alteração de imagem observada no exame de tomografia computadorizada demonstrou achado radiográfico relevante, com presença de radiopacidades no interior do seio maxilar decorrentes de sinusites fúngicas ou não fúngicas. O relato desse caso é relevante por apresentar alteração de imagem radiográfica exacerbada associada a quadros infecciosos agudos que podem comprometer o estado sistêmico do paciente imunossuprimido.

## INTRODUCTION

Calcification is a biochemical process in which deposition of calcium salts occurs; however, it may happen in unusual sites of human body.^1^ Pathological calcifications are classified as idiopathic, metastatic, dystrophic or intrasinus. These are called idiopathic when calcium builds up in healthy tissues but blood calcium level is normal. However, when blood tests positive calcium elevation with consequent ion deposition, metastatic calcification will be present. In dystrophic calcification, there is poor vascularization where calcium deposits, that is, not sufficient blood supply; in addition, necrotic tissues and ischemia may be seen on the site.^1^ It usually occurs in the core of growing tumors, where there is carbon dioxide decrease and extracellular fluid alkalinity increase, resulting in a microenvironment in which calcium is easily deposited. Intrinsic calcification derives from inflammatory and infectious conditions.^2^

The liver is the main organ for intermediate metabolism of proteins, carbohydrates, and fats as it metabolizes and excretes toxic substances. Chronic liver disease may alter these functions, especially in the presence of a perinatal inflammatory process initiated in bile ducts, resulting in progressive fibrosclerosis and intra- and extrahepatic obstruction.^3^ ^,^ ^4^ ^,^ ^5^ Hepatic transplantation is often the preferred therapy for a wide range of chronic liver diseases.^6^ After transplantation, calcineurin inhibitors such as cyclosporine and tacrolimus are initiated, which dramatically increases the transplanted organ’s lifetime.^6^ Some oral manifestations are relevant and specific to pediatric patients with this systemic disease. Color change in dental enamel and soft tissues is one of them, in both cases presenting greenish pigmentation, as well as enamel hypoplasias, eruption delay, and increased volume of pulp chamber and root canals.^3^ In order to analyze such alterations, volumetric computed tomography is often required, as it is classified as the best available method to evaluate hard-tissue lesions, especially in the mandible regions.^7^ ^,^ ^8^

Thus, the aim of this paper is to describe the case of a pediatric patient with dystrophic calcifications in maxillary sinus and bilirubin dental pigmentations after liver transplantation.

## CASE DESCRIPTION

A female patient, 12 years and 9 months old, presented for dental treatment at Universidade Federal de São Paulo (Unifesp), complaining of color change in dental enamel. The patient had been born of 40 weeks, by C-section, with intense neonatal jaundice. Even after phototherapy for three days, the condition showed no remission. At three years of age, she was diagnosed with biliary atresia, and liver transplantation was performed at the age of seven years and 11 months. The immunosuppressive medication administered was tacrolimus, with 1 mg in the morning and 1 mg in the evening.

**Figure 1:**
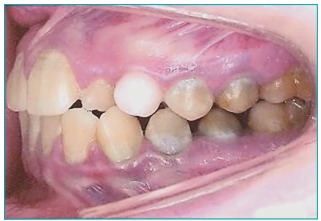
Intraoral view with prolonged retention of left superior deciduous canine and greenish pigmentation of teeth.

**Figure 2:**
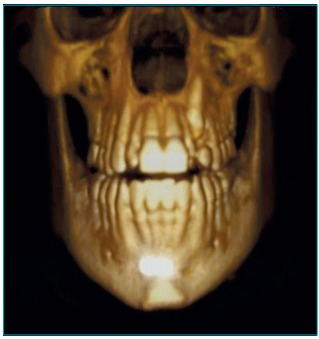
Frontal three-dimensional image.

**Figura 3:**
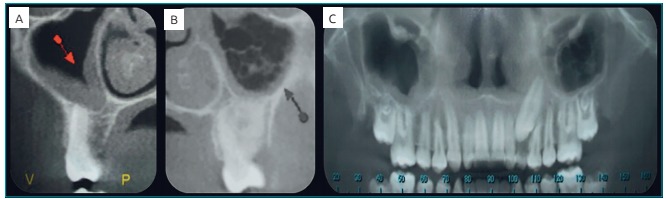
(A) Sagittal view showing peripheral dystrophic calcification of maxillary sinus; (B) Sagittal view showing peripheral dystrophic calcification of maxillary sinus and onset of new calcification areas in its center; (C) Panoramic view showing dystrophic calcifications of maxillary sinus.

Mixed dentures with greenish pigmentation in dental elements, as well as dental biofilm and prolonged retention of the left superior deciduous canine were observed upon intraoral examination (Figure 1). Cone beam CT scan of the maxilla was performed at *Centro de Tomografia Avançada* (CTA). The equipment used was an I-CAT (Kavo^•^) with cone-beam X-ray system, focal point of 0.5 mm, voxel of 0.125 mm, 14-bit gray scale, 17x23 cm field of view (FOV), automatic collimation with pulsed exposure, effective dose of 36 to 74 µSv and cylindrical reconstruction. The method consisted of a single exposure using cone-beam X-ray, capturing an image of the whole volume with a single exposure and 360° rotation of x-ray source around the patient’s head. To perform the tomographic report on the different radiographic densities of pigmented teeth, the Hounsfield scale was used in 16 shades, from light gray to black. CT scan showed prolonged retention of the left superior deciduous canine (Figure 2) in frontal three-dimensional view, and sagittal sections showed peripheral hyperdense images of maxillary sinus (Figure 3). Upon panoramic examination, peripheral dystrophic calcifications of maxillary sinus were seen (Figure 3).

## DISCUSSION

Dystrophic calcification in maxillary sinus originates from an inflammatory picture with chronicity characteristics and may be related to fungal or non-fungal sinusitis. Intrasinus calcium deposits may arise along with non-fungal inflammatory processes such as presence of mucocele and bacterial sinusitis.^2^ Few reports in literature mention non-fungal sinusitis, and differential diagnosis between both conditions is relevant.

The present report shows absence of differences in radiographic density of pigmented teeth in relation to enamel organ, without color change after cone-beam volumetric CT scan. This method allows examining the human body in segments with few millimeters of thickness, which helps to diagnose pathologies that affect bone tissues, besides being non-invasive, fast and of high diagnostic accuracy, being able to identify and delineate pathological processes.^9^ Computed tomography is currently used for oral rehabilitation, delimitation, and visualization of maxillofacial pathologies, but it is still little used to diagnose head and neck systemic pathologies.^9^

At images, the pigmented dental structure did not show radiographic density difference, suggesting that dental organs with bilirubin chromatic alterations in their structure can be submitted to restorative treatment. This pigmentation is a consequence of the high concentration of bilirubin in the dentinal tubules.^3^ Tomographic imaging also showed dystrophic calcifications inside the maxillary sinus, with peripheral areas of hyperdensity and hypodense center. The images suggest non-fungal sinusitis, in which calcification is close to the thickened submucosal layer of the maxillary sinus, constantly affected by chronic inflammatory conditions.^2^ Other etiologies cited in relation to this radiographic finding are inflammatory diseases, malignant tumors and benign lesions, mucoceles, and bacterial sinusitis.^2^ Calcifications in fungal sinusitis occur in the core of maxillary sinus, with hyperdensity originating from well-delineated nodular masses arising from the calcium depositions within mycelial mass.^2^ There is no consensus on the level of thickening of the sinus mucosa that is considered abnormal, ranging from 2 to 6 mm.^10^ The radiographic pattern of nonfungal sinusitis differs from fungal sinusitis, which is characterized by high density in maxillary sinus, bone destruction, and infiltration of adjacent soft tissue, allowing aggravating processes that may lead to death in immunosuppressed patients.^2^ In this case, a patient with liver transplantation may present serious clinical implications, once the adequate treatment of infections and sinusitis requires reduction or complete elimination of immunosuppression. If low immunosuppression continuation is needed, transplant rejection may occur.

Another factor to be highlighted in this case is the absence of symptoms, although dental pathological processes may potentially induce inflammation in maxillary sinus because of the proximity of dental floor to the radicular portion.^10^ The patient had healthy teeth, which shows no relationship between calcification and pathological dental processes. Patients with acute sinusitis usually report unilateral headache and maxillary algesia in dental region, with sensitive and painful teeth; facial edema and thick purulent secretion may occur in chronic sinusitis.^11^ This finding underlines the importance of conducting a cone-beam CT scan to diagnose orofacial pathologies.^10^

Along with results obtained by imaging evaluation, a tailored treatment plan was developed based on weekly dental prophylaxis with oral hygiene guidance and dental biofilm disclosure in order to avoid the installation of a gingival inflammatory process and the onset of incipient caries lesions. Later on, the deciduous canine was extracted.

Therefore, it can be inferred that imaging was fundamental for diagnosis of non-fungal sinusitis in immunosuppressed and asymptomatic patient, which allowed the medical team to start the treatment for a disease that can aggravate the overall condition of liver transplantation recipient.
